# Noninvasive Assessment of Intracranial Pressure: Deformability Index as an Adjunct to Optic Nerve Sheath Diameter to Increase Diagnostic Ability

**DOI:** 10.1007/s12028-024-01955-x

**Published:** 2024-03-06

**Authors:** Dag Ferner Netteland, Mads Aarhus, Else Charlotte Sandset, Llewellyn Padayachy, Eirik Helseth, Reidar Brekken

**Affiliations:** 1 Department of Neurosurgery, Oslo University Hospital, Pb 4956 Nydalen, 0424 Oslo, Norway; 2 Faculty of Medicine, University of Oslo, Oslo, Norway; 3 Department of Neurology, Oslo University Hospital, Oslo, Norway; 4 The Norwegian Air Ambulance Foundation, Oslo, Norway; 5 Department of Neurosurgery, School of Medicine, Faculty of Health Sciences, University of Pretoria and Steve Biko Academic Hospital, Pretoria, South Africa; 6 Department of Health Research, Medical Technology, SINTEF, Trondheim, Norway

**Keywords:** Deformability index, Optic nerve sheath diameter, Ultrasound, Noninvasive, Intracranial pressure, Traumatic brain injury

## Abstract

**Background:**

Today, invasive intracranial pressure (ICP) measurement remains the standard, but its invasiveness limits availability. Here, we evaluate a novel ultrasound-based optic nerve sheath parameter called the deformability index (DI) and its ability to assess ICP noninvasively. Furthermore, we ask whether combining DI with optic nerve sheath diameter (ONSD), a more established parameter, results in increased diagnostic ability, as compared to using ONSD alone.

**Methods:**

We prospectively included adult patients with traumatic brain injury with invasive ICP monitoring, which served as the reference measurement. Ultrasound images and videos of the optic nerve sheath were acquired. ONSD was measured at the bedside, whereas DI was calculated by semiautomated postprocessing of ultrasound videos. Correlations of ONSD and DI to ICP were explored, and a linear regression model combining ONSD and DI was compared to a linear regression model using ONSD alone. Ability of the noninvasive parameters to distinguish dichotomized ICP was evaluated using receiver operating characteristic curves, and a logistic regression model combining ONSD and DI was compared to a logistic regression model using ONSD alone.

**Results:**

Forty-four ultrasound examinations were performed in 26 patients. Both DI (*R* =  − 0.28; 95% confidence interval [CI] *R* <  − 0.03; *p* = 0.03) and ONSD (*R* = 0.45; 95% CI *R* > 0.23; *p* < 0.01) correlated with ICP. When including both parameters in a combined model, the estimated correlation coefficient increased (*R* = 0.51; 95% CI *R* > 0.30; *p* < 0.01), compared to using ONSD alone, but the model improvement did not reach statistical significance (*p* = 0.09). Both DI (area under the curve [AUC] 0.69, 95% CI 0.53–0.83) and ONSD (AUC 0.72, 95% CI 0.56–0.86) displayed ability to distinguish ICP dichotomized at ICP ≥ 15 mm Hg. When using both parameters in a combined model, AUC increased (0.80, 95% CI 0.63–0.90), and the model improvement was statistically significant (*p* = 0.02).

**Conclusions:**

Combining ONSD with DI holds the potential of increasing the ability of optic nerve sheath parameters in the noninvasive assessment of ICP, compared to using ONSD alone, and further study of DI is warranted.

**Supplementary Information:**

The online version contains supplementary material available at 10.1007/s12028-024-01955-x.

## Introduction

The assessment and control of intracranial pressure (ICP) is an essential part of the management of several neurological conditions, including traumatic brain injury (TBI) [1]. In today’s clinical practice, measuring ICP invasively remains the standard. However, invasive ICP monitoring is associated with certain drawbacks. Its demand on resources confers limitations on its availability in settings where resources are constrained. Additionally, its invasive nature makes it unsuitable for early diagnostic triage, and it also harbors small risks of infection and hemorrhage [2]. This warrants a pursuit of a quick, reliable, and widely available noninvasive method of ICP measurement.

Optic nerve sheath diameter (ONSD) has emerged as a parameter for noninvasive ICP assessment. Physiologically, the cerebrospinal fluid (CSF) enveloped by the optic nerve sheath is directly communicating with the intracranial CSF. Hence, increases in ICP leads to distention of the optic nerve sheath. This sheath can be visualized and its diameter can be measured by transorbital ultrasound, making the method available at the bedside. Indeed, studies have shown a promising association between ONSD and ICP [3]. However, to increase clinical relevance, the method is still in need of further refinement to increase its diagnostic accuracy.

**Fig. 1:**
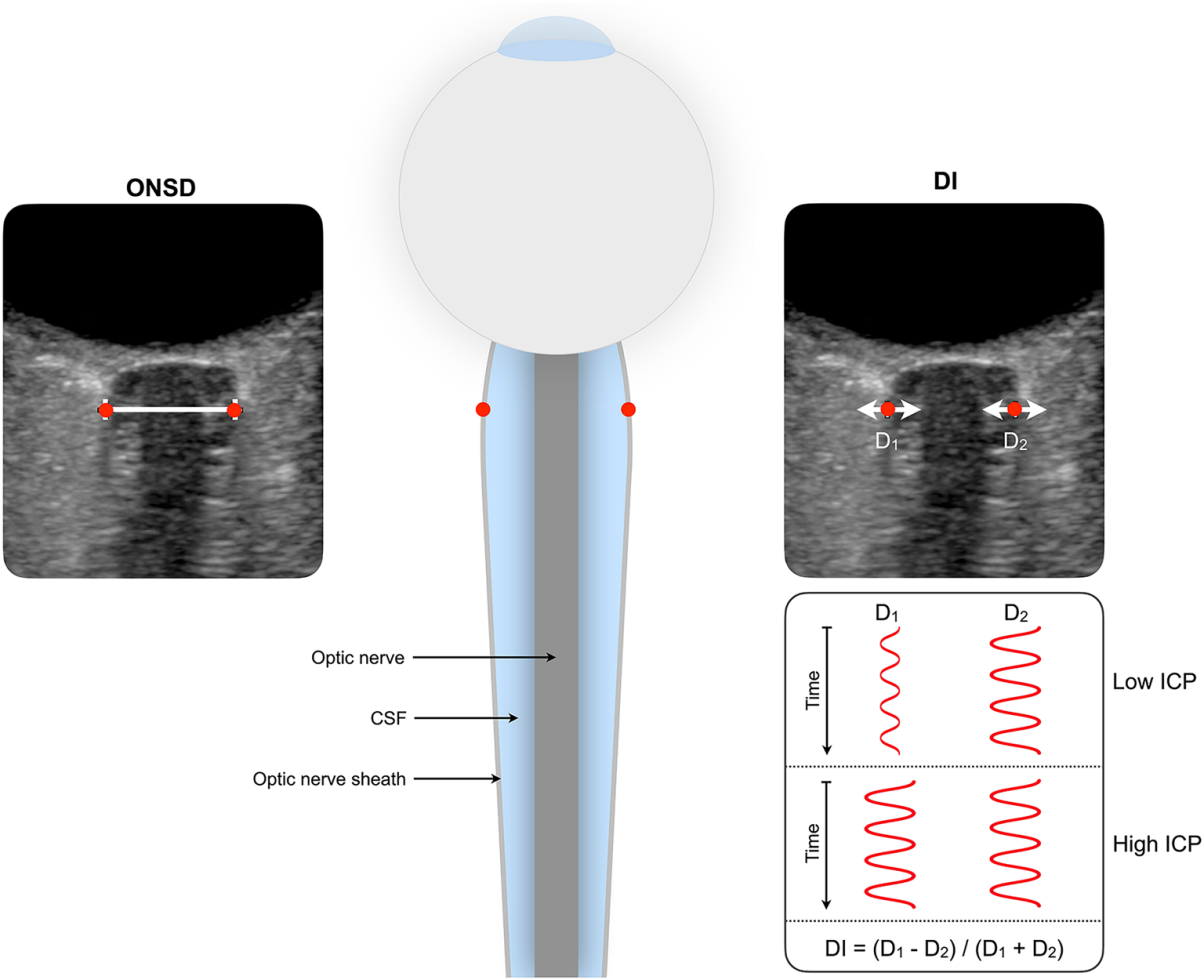
Illustrative figure of the optic nerve sheath complex (middle) with ultrasound images depicting ONSD (left) and DI (right). Red dots are placed on the optic nerve sheath in both the illustrative figure and the ultrasound images. ONSD is the distance between the two opposite points on the optic nerve sheath 3 mm posterior to the retina measured on a still image. DI is a dynamic parameter based on the same two points on the optic nerve sheath but uses automatic speckle tracking to identify the lateral movement of these points on ultrasound videos. DI estimates the magnitude of motion in the lateral direction on both sides of the nerve sheath complex (D_1_ and D_2_) over the cardiac cycle and quantifies the deformation of the nerve sheath according to the formula DI = (D_1_ − D_2_) / (D_1_ + D_2_). The proposed concept hypothesizes that the lateral movement of D_1_ and D_2_ is asymmetrical when ICP is low and becomes more symmetrical when ICP is high. CSF cerebrospinal fluid, DI deformability index, ICP intracranial pressure, ONSD optic nerve sheath diameter (Color figure online)

The deformability index (DI) is a novel dynamic optic nerve sheath parameter. It describes the relative lateral motion of opposite sides of the optic nerve sheath occurring with each cardiac cycle and hypothesizes that this relative motion is nonsymmetrical in states of low ICP and becomes more symmetrical in states of high ICP (Fig. 1). DI can be obtained by processing of ultrasound videos of the optic nerve sheath and can, like ONSD, potentially be available at the bedside.

The first studies evaluating DI [4, 5] demonstrated that it correlated with ICP and was able to distinguish dichotomized ICP in two pediatric populations in which hydrocephalus was the predominant underlying condition. Moreover, the results gave an indication that combining ONSD and DI may increase diagnostic ability over using either parameter alone.

In the present study, the first to evaluate DI in an adult population, we test its ability to assess ICP noninvasively in patients with TBI. Furthermore, we ask whether combining ONSD and DI will increase diagnostic ability as opposed to using ONSD alone.

## Methods

In this study, we prospectively included adult patients with TBI who were admitted to the neurointensive care unit at Oslo University Hospital between January 2020 and August 2022. The reference method of measuring ICP, to which the noninvasive determinants were compared, was invasive monitoring, measured either parenchymally or intraventricularly. Transorbital ultrasound images and videos of the optic nerve sheath complex were acquired by a single experienced ultrasound operator (DFN). ONSD was measured manually at the bedside by the operator, whereas DI was calculated by semiautomated postprocessing of ultrasound videos by an investigator (RB) blinded to the invasively measured ICP at the time of processing. Patients were examined by transorbital ultrasound and were thereby included in the study, as per availability of the ultrasound operator.

Included patients were treated according to our institutional TBI management protocol, concordant with Brain Trauma Foundation guidelines [6], and inclusion in the study did not interfere with standard management. As per this protocol, maintaining ICP at ≤ 22 mm Hg was a main aim of management. Nonsurgical ICP-lowering measures included elevation of the head of the bed to 30 degrees, deep sedation, hypertonic saline, normocapnia, and thermoregulation to normothermia. Surgical ICP-lowering measures included CSF drainage and craniotomy with mass lesion evacuation. Last-tier measures included decompressive craniectomy and thiopental sedation to burst suppression.

In accordance with the Helsinki Declaration [7], proxy consent was obtained for unconscious patients at the time of inclusion. If the patient regained ability to give informed consent, this was obtained from the patient at a later stage. The study was approved by the Regional Committee for Medical and Health Research Ethics South East Norway (2018/136).

### Patient eligibility

Adult patients (≥ 18 years old) with head CT scan findings consistent with TBI who were admitted to the neurointensive care unit with invasive ICP monitoring were eligible for inclusion. Invasive ICP monitoring was required to be recorded via a correctly positioned and functioning parenchymal sensor or an external ventricular drain. In patients with intraparenchymal ICP sensors, ultrasound data sets were acquired within 7 days of implantation to avoid significant drift from zero in the invasively measured ICP readings [8]. Where invasive measurements were performed via an external ventricular drain, no limitation to the in situ time was adhered to. Patients with unilateral or bilateral injuries to the orbital region were excluded from the study.

### Data Acquisition

Transorbital ultrasound images and videos were acquired using a commercially available scanner (Philips Epiq 5G; Philips Healthcare, Andover, MA) with a linear array probe (Philips L14-7io). Established safety margins of ophthalmic ultrasound imaging were adhered to, with a mechanical index of less than 0.23. Serial measurements in the same patient were obtained at opportunity.

ONSD was measured at the bedside by manually identifying the optic nerve sheath 3 mm posterior to the retina in one plane. Ultrasound videos (10 s) for DI calculation were acquired after ONSD measurement for each eye by holding the probe still with the optic nerve sheath in focus. Invasive ICP was measured using either a parenchymal microsensor (Codman microsensor; Johnson and Johnson, Raynham, MA, or Raumedic Neurovent-*P* ICP sensor; Raumedic AG, Münchberg, Germany) or a ventricular catheter and was recorded at the time of the ultrasound examination.

Additional parameters, including physiological parameters (e.g., pulse rate, blood pressure etc.), Richmond Agitation-Sedation Scale score, details on surgical management, and the presence of CSF drainage or leak, were also acquired at the time of the ultrasound examination.

### Deformability Index

DI was calculated according to the method previously described by our research group [4, 5]. It estimates the magnitude of motion in the lateral direction on both sides of the optic nerve sheath (D1 and D2) over the cardiac cycle (Fig. 1) and quantifies the deformation of the nerve sheath complex according to the formula DI = (D1 − D2) / (D1 + D2). In this study, manual initialization of two opposite points on the optic nerve sheath was used before automatic speckle tracking of the lateral motion of the points was performed. The depth of initialization was standardized at 3 mm from the sclera. To reduce variability from the manual initialization, DI was processed three times, and resultant values were averaged for each eye. Averaged values between the two eyes were used for final analysis.

### Image Quality Categorization

For each ultrasound video, the image quality was independently evaluated by two investigators (DFN and RB). A label of “adequate” or “too poor” image quality was applied to (1) the quality of the optic nerve sheath visualization and (2) the presence of movement artifacts. Discrepancies in “too poor” labels were reviewed to reach consensus. Examinations in which at least one quality parameter was labeled “too poor” after consensus for at least one of the eyes were categorized as “red.” Remaining examinations were categorized as “green,” representing adequate image quality.

### End Points

End points and planned subgroup analyses were specified per study protocol prior to commencement of data collection. Primary end points were (1) correlation between DI, ONSD, or the combination of the two and ICP and (2) the ability of DI, ONSD, or the combination of the two to distinguish dichotomized ICP. Planned subgroup analyses included subgrouping by image quality score and by open versus closed skull vault. The latter was based on a hypothesis that any opening of the enclosed skull vault to the outside atmospheric pressure (decompressive craniectomy, CSF fistula/leak) or external resistance (open external ventricular or lumbar drain) may impact the physiology underlying DI and relates to the principles of the Monro-Kellie doctrine [9].

### Data Analysis

Correlations between ONSD and ICP and between DI and ICP were explored using the Pearson correlation coefficient *R*. Based on previous work, a positive correlation between ONSD and ICP and a negative correlation between DI and ICP was hypothesized. Correlations were therefore tested with a one-tailed *t*-test, and results reported with a one-sided 95% confidence interval (CI). Simple linear regression was used to study ICP as a function of ONSD or DI, and multiple linear regression was used for the combination of ONSD and DI. The coefficient of determination (*R*^2^) for the combined model was compared to that of the model predicting ICP based on ONSD alone, and the difference between the models was evaluated using analysis of variance.

To compare the two noninvasive parameters’ ability to distinguish dichotomized ICP, a cutoff at ICP ≥ 15 mm Hg was chosen. Additionally, cases were also dichotomized at an ICP of ≥ 20 mm Hg. For both dichotomizations, median ONSD and DI were calculated for each group and compared using a one-tailed Mann–Whitney *U*-test. Receiver operating characteristic (ROC) analysis was used to calculate associated areas under the curve (AUCs) with 95% CIs. Logistic regression was used to study the combination of ONSD and DI for predicting dichotomized ICP, and analysis of variance was used to compare this model to a logistic regression using ONSD alone.

Preplanned subgroup analyses by image quality score and open vs. closed cranial vault were performed by examining correlations between ICP and DI using Pearson correlation coefficients with one-sided 95% CIs in resultant subgroups. To further characterize potential physiological reasons for correlations between ICP and DI, we also performed exploratory analysis evaluating correlation between ICP amplitude and DI. Statistical analyses were performed using Matlab (R2022a; Mathworks Inc, Natick, MA), and *p* < 0.05 was considered significant.

## Results

### Patient Characteristics

A total of 26 adult patients with TBI were included in the study; 20 (77%) patients were male, and 6 (23%) patients were female. The median age was 55 years (interquartile range [IQR] 35–65 years). All patients sustained a blunt head injury, and the most common mechanism of injury was falls (38%), followed by motor vehicle accidents (15%) and sports and recreational injuries (15%). The median initial Glasgow Coma Scale score [10] was 7 (IQR 3–11). All patients were intubated and sedated, and the median Richmond Agitation-Sedation Scale score at the time of examination was − 5 (IQR − 4 to − 5).

### Optic Nerve Sheath Examinations and ICP

A total of 44 ultrasound examinations were performed in the 26 included patients. Serial ultrasound examinations were performed in 13 of 26 (50%) patients, and the median number of examinations in those with serial examinations was 2 (range 2–4). The median time between serial examinations was 1 day (IQR 1–2 days; range 0–12 days). Invasive ICP was recorded at the time of the ultrasound examination. The recorded ICP values ranged between 5 and 23 mm Hg (median 14 mm Hg, IQR 9–17 mm Hg).

### Correlation

**Fig. 2:**
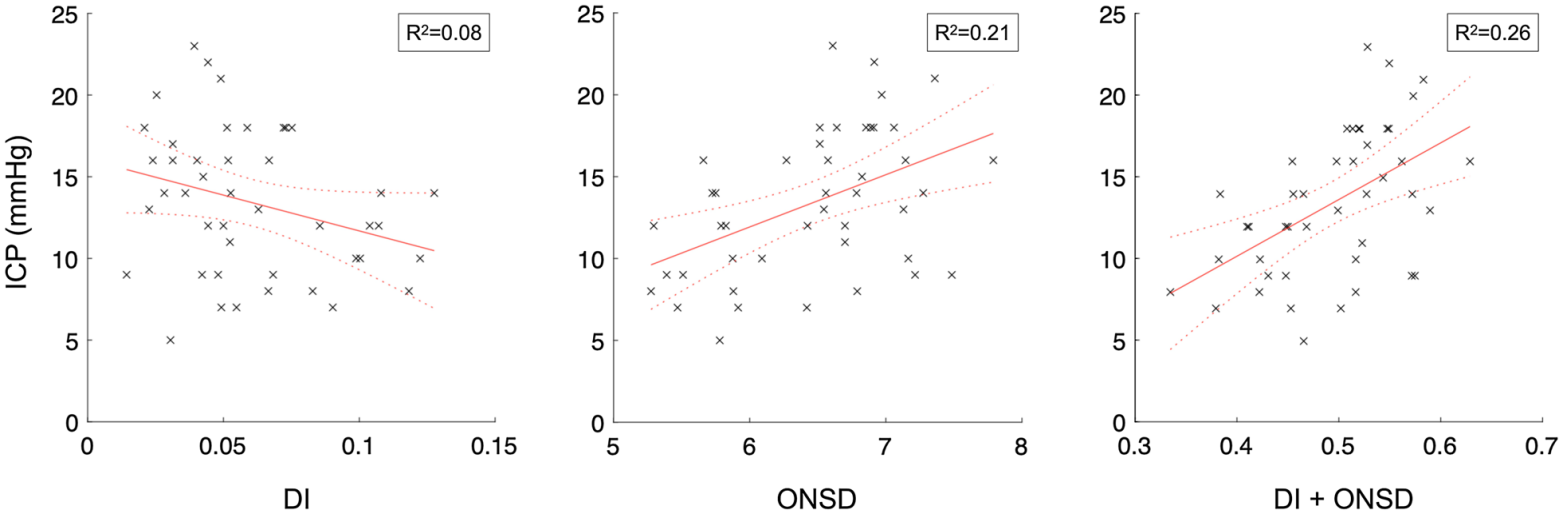
Correlation between DI/ONSD and ICP. Scatterplots with linear regression lines, associated confidence bounds, and *R*^2^ values for DI (left), ONSD (middle), and the combination of ONSD and DI (right). DI deformability index, ICP intracranial pressure, ONSD optic nerve sheath diameter

There was a negative linear correlation between DI and ICP (*R* =  − 0.28; 95% CI *R* <  − 0.03; *p* = 0.03) and a positive linear correlation between ONSD and ICP (*R* = 0.45; 95% CI *R* > 0.23; *p* < 0.01). When including ONSD and DI in a multiple linear regression, the estimated correlation coefficient increased (*R* = 0.51; 95% CI *R* > 0.30; *p* < 0.01), compared to using ONSD alone. However, the model improvement did not reach statistical significance (*p* = 0.09). The linear regressions with associated *R*^2^ values are shown in Fig. 2.

### Ability To Distinguish Dichotomized ICP

#### *ICP* ≥ *15 mm Hg Cutoff*

**Fig. 3:**
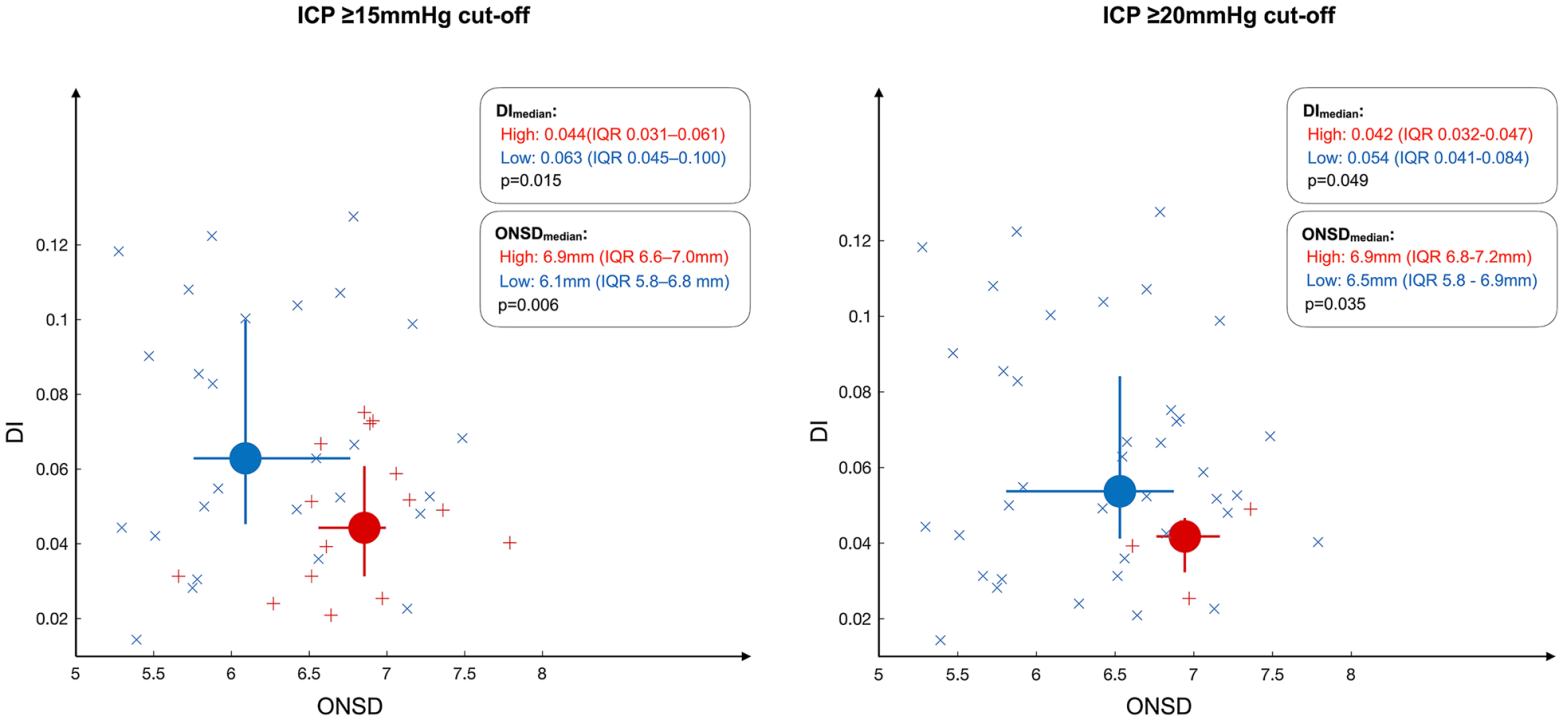
Ability of DI and ONSD to distinguish dichotomized ICP. Classification plot showing ONSD and DI for all examinations dichotomized into low ICP (x) and high ICP ( +). Dots illustrate the median for each group, whereas bars indicate the IQR. Left: ICP dichotomization at ≥ 15 mm Hg. Right: ICP dichotomization at ≥ 20 mm Hg. DI deformability index, ICP intracranial pressure, IQR interquartile range, ONSD optic nerve sheath diameter

**Fig. 4:**
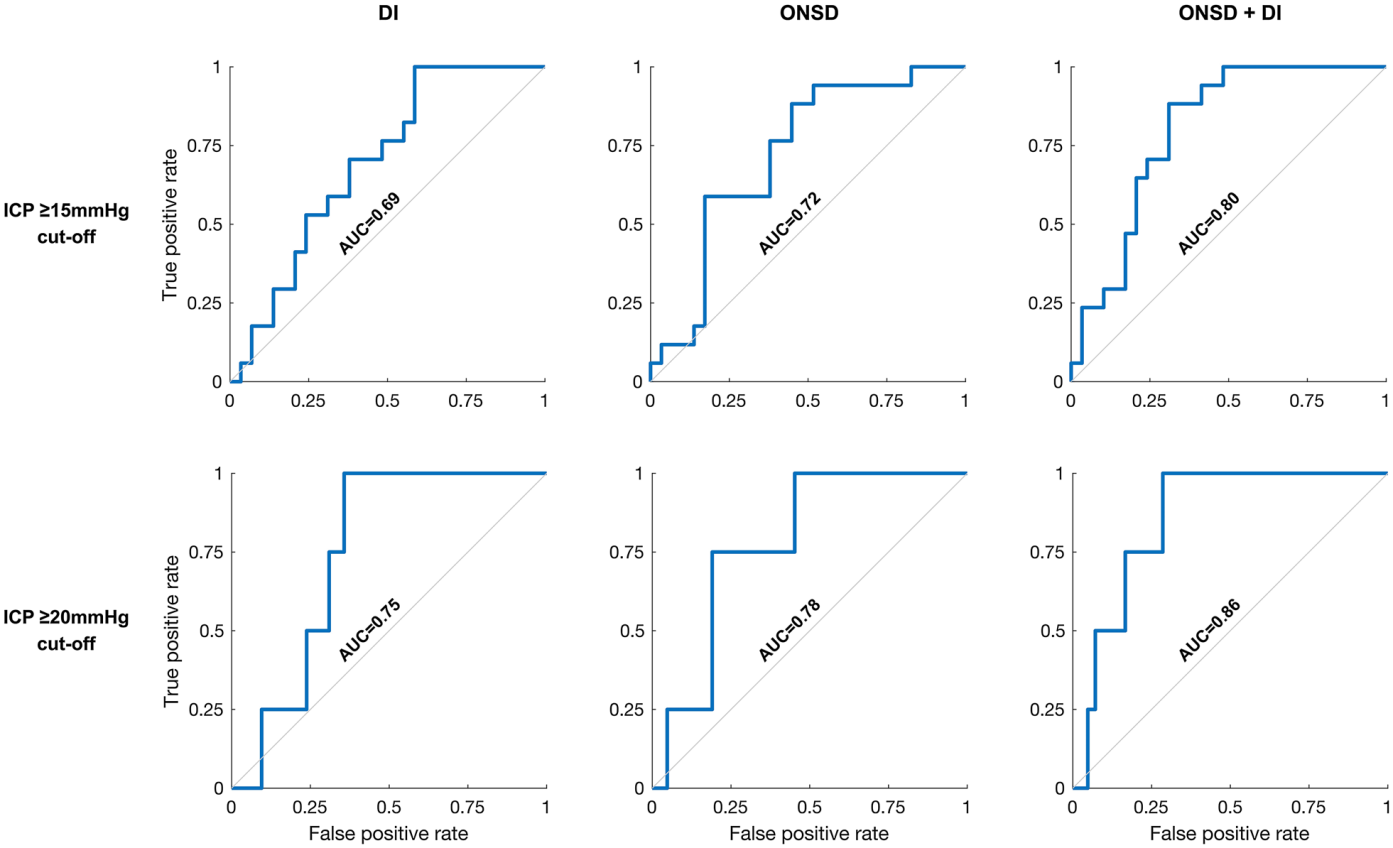
Ability of DI, ONSD, or the combination of the two to distinguish dichotomized ICP. Receiver operating characteristic curves. Top: ICP dichotomized at ≥ 15 mm Hg. Bottom: ICP dichotomized at ≥ 20 mm Hg. Left: DI. Middle: ONSD. Right: ONSD and DI combined. AUC area under the curve, DI deformability index, ICP intracranial pressure, ONSD optic nerve sheath diameter

Analysis by dichotomization at ICP ≥ 15 mm Hg (high ICP ≥ 15 mm Hg: *n* = 27; low ICP < 15 mm Hg: *n* = 17) showed a significant difference in both median DI and ONSD between the groups (Fig. 3). For DI alone, the AUC was 0.69 (95% CI 0.53–0.83), whereas the AUC for ONSD alone was 0.72 (95% CI 0.56–0.86). When including DI and ONSD in a combined model, the estimated AUC increased (0.80, 95% CI 0.63–0.90) as compared to using ONSD alone, and when comparing the models, the combined model distinguished dichotomized ICP significantly better than the model using ONSD alone (*p* = 0.02). ROC curves are shown in Fig. 4.

#### *ICP* ≥ *20 mm Hg Cutoff*

For an ICP threshold of ≥ 20 mm Hg (high ICP ≥ 20 mm Hg: *n* = 4; low ICP < 20 mm Hg: *n* = 40), both median DI and ONSD were significantly different between groups (Fig. 3). ROC analysis gave an AUC of 0.75 (95% CI 0.58–0.90) for DI alone and an AUC of 0.78 (95% CI 0.52–0.93) for ONSD alone. When including DI and ONSD in a combined model, the estimated AUC increased to 0.86 (95% CI 0.67–0.96), as compared to when using ONSD alone. However, the model improvement did not reach statistical significance (*p* = 0.11). ROC curves are shown in Fig. 4.

### Subgroup Analyses

#### By Image Quality Score

The initial agreement between the two authors in evaluating quality as either “too poor” or “adequate” in the two evaluation categories was 97% (170 of 176). After consensus, seven examinations were categorized as “red,” and the remaining 39 examinations were categorized as “green.”

**Fig. 5:**
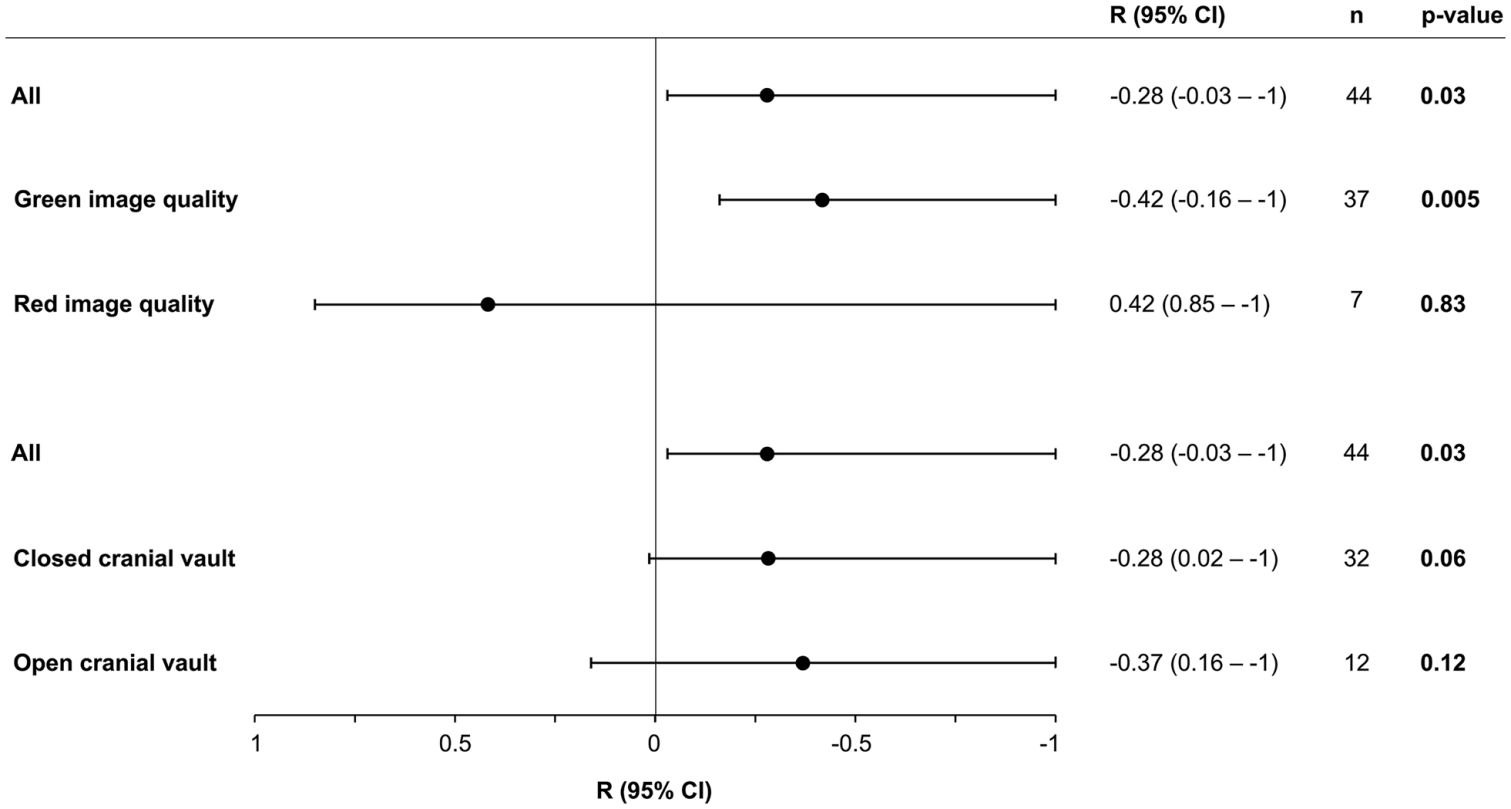
Subgroup analyses. Forest plot of Pearson *R* values and associated one-sided 95% CIs and *p* values for correlation between DI and ICP for subgroups by image quality and open vs. closed cranial vault. CI confidence interval, DI deformability index, ICP intracranial pressure

Analyses by image quality score showed that when including only examinations in which image quality was deemed adequate (green image quality), the correlation between DI and ICP increased as compared to that of all examinations. Conversely, in the red image quality group (image quality deemed too poor), we found no correlation between DI and ICP. Results are shown in Fig. 5.

When repeating the comparison between the combined regression model including both DI and ONSD and the model using ONSD alone from the primary analysis, but with only examinations of green image quality included, the combined model now correlated significantly better to ICP compared to the model using ONSD alone (*p* = 0.03).

#### By Open vs. Closed Skull Vault

Decompressive craniectomy, active CSF drainage, and CSF leak were considered as factors potentially modifying the physiology of the closed cranial vault, and one or more of these factors were present at the time of the ultrasound examination in 30% (14 of 46) of instances. When comparing correlations between DI and ICP in subgroups of open vs. closed skull vault, we detected no signal of this subgrouping significantly affecting correlations (Fig. 5). These analyses were performed to evaluate whether a correlation between DI and ICP potentially could be related to ICP amplitude, which typically also increases with increasing ICP. However, concordant with the results from these subgroup analyses, exploratory analysis of correlation between DI and ICP amplitude did not show a significant correlation between the two (*R* =  − 0.13, 95% CI *R* < 0.13; *p* = 0.20).

## Discussion

In the present study, the first to evaluate DI in an adult population, we show that DI individually correlated with ICP and displayed ability in distinguishing dichotomized ICP. When combining DI with the more established ONSD, there was a tendency toward increased correlation, but the model improvement did not reach statistical significance. However, a combined model using both parameters did display significantly better ability in distinguishing dichotomized ICP as compared to using ONSD alone. This is congruent with previously published results from pediatric series [4, 5]. These study populations differ in important aspects from the one of the current study. For one obviously in age, but also in the underlying condition leading to raises in ICP, as in the pediatric series the majority of patients suffered from more chronic underlying conditions such as hydrocephalus. Importantly, the results of the present study indicate that the concept of DI is not limited to children or ICP elevations of a more long-standing nature but instead also extends to adults and more acute increases in ICP, such as those associated with TBI.

Our study has several important limitations. First, the overall number of observations are limited. Furthermore, the number of observations in the higher ICP ranges are limited. The latter is related to the patient group studied, as once invasive ICP monitoring is established, a main goal of neurointensive management of TBI is to control ICP below a certain threshold.

The currently recommended ICP treatment threshold in the Brain Trauma Foundation guidelines is 22 mm Hg, and this is reflected in the institutional management protocol according to which our patients were cared for [6]. However, this threshold has varied over the course of the previous three editions of the Brain Trauma Foundation guidelines [11–13], from 20 to 20–25 to 20 mm Hg, before the current recommendation was published in 2016. In a more recently published study using a large database of high-frequency ICP measurements, 19 mm Hg was found to be the threshold most robustly associated with mortality [14]. Hence, the ideal threshold at which ICP-lowering measures should be instigated on a group level is still generally considered somewhat uncertain [1].

Although the current guideline-recommended treatment threshold in TBI is 22 mm Hg, the upper limit of what constitutes normal ICP is considered lower by most, and although limits of < 10 mm Hg or < 15 mm Hg have been advocated for [14–16], the exact limit remains elusive [17]. Harm from intracranial hypertension is more likely situational, in which a number of factors (e.g., intactness of cerebrovascular autoregulatory capacity) probably will play a role, but the dose of intracranial hypertension (i.e., the magnitude and time over which intracranial hypertension is sustained) has been shown to be important [18, 19]. In this setting, data have suggested that sustained ICP > 15 mm Hg [19] and even > 10 mm Hg [14] can be associated with worse outcomes in patients with TBI.

To explore DI’s ability to distinguish dichotomized ICP and to compare the model combining DI and ONSD to a model using ONSD alone, we dichotomized our data by ICP cutoffs. To obtain a relative size balance between the two groups and because this has been a previously suggested limit of normal ICP, we chose to dichotomize at ICP at ≥ 15 mm Hg. Additionally, we performed analyses by dichotomization at 20 mm Hg, but these analyses remain statistically limited by only four observations in the high ICP group.

Although these cutoffs do not reflect the current guideline-recommended ICP management threshold, we believe that using these cutoffs still provides valid information on the discriminative ability of the noninvasive parameters as well as a valid comparison between the combined model using both parameters and the model using ONSD alone. Even so, the current study is limited to evaluating the noninvasive parameters’ correlation and discriminative ability in the range of ICP values of the included patients.

Secondly, DI was calculated by semiautomated postprocessing of ultrasound videos in the present study, whereas the method’s intended use is as a bedside parameter. This reflects the method’s stage of development during the inclusion phase of this study. In our study, two opposite points on the optic nerve sheath were manually initialized before automated speckle tracking of the motion of the points was performed. Ultimately, automatic identification of the opposite points on the optic nerve can make the method fully automated and hence make DI calculations available at the bedside at the time of measurement. These opposite points on the optic nerve sheath relate to the points between which ONSD is measured (Fig. 1). Indeed, the software for automatic identification of these points using real-time segmentation of the optic nerve sheath complex using machine learning has been developed and was tested on its ability to perform automated measurements of ONSD in a recently published study [20].

The same software also features a guidance system both regarding image alignment along the optic nerve axis to allow adequate visualization of the optic nerve sheath and providing real-time feedback to the investigator regarding probe movements. The components of the image quality score applied in the subgroup analysis mirror these guidance features. Indeed, when excluding acquisitions categorized as of too poor quality, we saw a clear increase in the correlation between DI and ICP, and the combined model using DI and ONSD now correlated significantly better to ICP compared to the model using ONSD alone.

Thirdly, ONSD and DI measurements were acquired after initialization of management interventions, whereas the most relevant potential use of the method may be seen as an early diagnostic tool. It can therefore be argued that the diagnostic ability of the noninvasive parameters ideally should be studied in the same preintervention phase. Logistically, this poses a challenge because of the need for a reference method of ICP measurements to which the noninvasive parameters are compared and the fact that invasive ICP monitoring typically is established as part of the initial acute surgical management. Ethically, it also poses a challenge because inclusion prior to initial management may then delay potentially lifesaving interventions, such as surgical decompression. Some of these interventions alter the physiological principles of the closed skull vault, and one can hypothesize that this may alter the physiological principles underpinning DI. Our subgroup analyses did not however support this notion by not indicating a difference in correlation between DI and ICP in subgroups by open versus closed skull vault.

Previous data have shown a reasonably good diagnostic accuracy of ONSD in determining ICP. In a meta-analysis of seven studies (320 patients) with invasive ICP measurements used as the reference [3], *R* values ranged between 0.50 and 0.76 in the included studies, and the calculated area under the hierarchical summary ROC curve was 0.94. Despite this, further refinement to better diagnostic accuracy is still desirable before noninvasive ICP assessment can be used to guide critical management decisions.

Here, we show that the diagnostic ability of ONSD can be improved on by combining DI with ONSD. DI is a dynamic parameter, and one can hypothesize that this can alleviate some of the challenges that likely exist with individual variation in baseline ONSD. It is also a parameter that is based on automated software. This holds the potential of providing automated simultaneous bedside measurements of both ONSD and DI, thereby alleviating some of the operator dependency that currently challenges transorbital ultrasound measurements. Given that these parameters continue to show ability in assessing ICP noninvasively in future studies, one could technologically envision a handheld ultrasound device providing automated measurements suited for clinicians and patients in a point-of-care setting.

## Conclusions

DI individually correlated with ICP and displayed ability in distinguishing dichotomized ICP. When combining DI with the more established ONSD, strength of correlation increased, but the model improvement did not reach statistical significance. However, a combined model using both parameters did display significantly better ability in distinguishing dichotomized ICP as compared to a model using ONSD alone. These findings warrant further study of DI as a parameter that holds potential in increasing the ability of optic nerve sheath parameters in the noninvasive assessment of ICP.

## Supplementary Information

Below is the link to the electronic supplementary material.

Supplementary file1 (PDF 200 kb)
