# Supplementary material for: Noninvasive Assessment of Intracranial Pressure: Deformability Index as an Adjunct to Optic Nerve Sheath Diameter to Increase Diagnostic Ability
Source: Neurocrit Care. 2024 Mar 6;41(2):479–88. doi: 10.1007/s12028-024-01955-x (PMC11377659; doi:10.1007/s12028-024-01955-x)
Supplement: Supplementary file 1 — Supplementary file1 (PDF 200 kb) [file 12028_2024_1955_MOESM1_ESM.pdf]

Supplementary table. Patient characteristics and ICP, ONSD and DI values

Patient#	Exam#	Age	Gender	Mechanism of Injury	Blunt vs Penetrating	Initial GCS	ICP	Δ ICP* (>2mmHg increase=blue; >2mmHg decrease=red)	ONSD	Δ ONSD (any increase=blue; any decrease=red)	DI	Δ DI (any decrease=blue; any increase=red)
1	1	33	M	Sports/recreational	B	7	21	NA	0,735	NA	0,049	NA
	2						18	-3	0,690	-0,045	0,072	0,023
	3						18	0	0,705	0,015	0,059	-0,013
	4						18	0	0,685	-0,020	0,076	0,017
2	1	41	F	Sports/recreational	B	12	12	NA	0,670	NA	0,107	NA
	2						16	4	0,715	0,045	0,052	-0,055
3	1	29	M	Other	B	3	7	NA	0,592	NA	0,055	NA
	2						7	0	0,642	0,050	0,049	-0,006
4	1	25	M	MVA	B	3	9	NA	0,722	NA	0,048	NA
	2						10	1	0,717	-0,005	0,099	0,051
	3						9	-1	0,749	0,032	0,069	-0,030
5	1	41	M	Fall	B	3	16	NA	0,779	NA	0,041	NA
6	1	62	M	Unknown	B	12	8	NA	0,588	NA	0,083	NA
7	1	71	F	MVA	B	3	5	NA	0,578	NA	0,031	NA
	2						10	5	0,588	0,010	0,123	0,092
8	1	79	M	Unknown	B	15	13	NA	0,713	NA	0,023	NA
9	1	65	F	Fall	B	9	7	NA	0,547	NA	0,090	NA
10	1	64	F	MVA	B	3	12	NA	0,583	NA	0,050	NA
11	1	72	M	Fall	B	3	16	NA	0,566	NA	0,032	NA
	2						12	-4	0,530	-0,036	0,045	0,013
12	1	69	M	Fall	B	7	10	NA	0,609	NA	0,101	NA
	2						16	6	0,658	0,049	0,067	-0,034
	3						11	-4	0,670	0,012	0,052	-0,015
13	1	75	F	Fall	B	9	14	NA	0,656	NA	0,036	NA
	2						15	1	0,683	0,027	0,043	0,007
14	1	59	M	Sports/recreational	B	6	12	NA	0,643	NA	0,104	NA
	2						14	2	0,679	0,036	0,128	0,024
15	1	18	M	MVA	B	3	14	NA	0,573	NA	0,108	NA
	2						17	3	0,652	0,079	0,032	-0,076
16	1	33	M	Other	B	5	18	NA	0,691	NA	0,073	NA
	2						22	4	0,692	0,001	0,045	-0,028
	3						20	-2	0,697	0,005	0,026	-0,019
17	1	61	M	Fall	B	13	16	NA	0,627	NA	0,024	NA
18	1	66	M	Fall	B	3	9	NA	0,551	NA	0,042	NA
19	1	21	M	MVA	B	7	14	NA	0,728	NA	0,053	NA
20	1	61	F	Sports/recreational	B	6	18	NA	0,664	NA	0,021	NA
21	1	58	M	MVA	B	14	9	NA	0,539	NA	0,015	NA
22	1	39	M	Unknown	B	11	23	NA	0,661	NA	0,039	NA
23	1	36	M	Fall	B	13	8	NA	0,528	NA	0,119	NA
24	1	49	M	Fall	B	4	14	NA	0,575	NA	0,028	NA
	2						12	-2	0,579	0,004	0,086	0,058
25	1	52	M	Fall	B	8	13	NA	0,655	NA	0,063	NA
	2						18	5	0,652	-0,003	0,051	-0,012
26	1	37	M	Other	B	3	8	NA	0,679	NA	0,067	NA

GCS=Glasgow Coma Scale. ICP=intracranial pressure. ONSD=optic nerve sheath diameter. DI=deformability index. Δ=change from previous recorded value. Blue coloured boxes annotates a decrease in ICP >2mmHg from previous value, any decrease in ONSD and any increase in DI. Red coloured boxes annotates a decrease in ICP >2mmHg from previous value, any decrease in ONSD and any increase in DI. *Where serial examinations were performed in the same patient, examinations were performed in different days.
